# Poly(I:C)-Induced Mesenchymal Stem Cells Protect the Kidney Against Ischemia/Reperfusion Injury *via* the TLR3/PI3K Pathway

**DOI:** 10.3389/fmed.2021.755849

**Published:** 2021-11-25

**Authors:** Tian Chen, Yamei Jiang, Shihao Xu, Yin Celeste Cheuk, Jiyan Wang, Cheng Yang, Ruiming Rong

**Affiliations:** ^1^Department of Urology, Zhongshan Hospital, Fudan University, Shanghai, China; ^2^Shanghai Key Laboratory of Organ Transplantation, Shanghai, China; ^3^Department of Urology, Shanghai Public Health Clinical Center, Shanghai, China

**Keywords:** acute kidney injury, renal ischemia-reperfusion injury, mesenchymal stem cell, polyinosinic-polycytidylic acid, anti-inflammatory phenotype

## Abstract

**Objective:** To investigate the effect and protective mechanism of mesenchymal stem cell subpopulations on acute kidney injury by establishing a mouse model of renal ischemia-reperfusion injury.

**Methods:** Male C57BL/6 mice were randomly divided into five groups, namely, sham-operation group and those treated with normal saline, untreated mesenchymal stem cells, mesenchymal stem cells treated with lipopolysaccharide (LPS, pro-inflammatory phenotype) and mesenchymal stem cells treated with polyinosinic-polycytidylic acid (poly[I:C], anti-inflammatory phenotype) respectively. The renal function, histopathological damage, circulating inflammation levels and antioxidant capacity of mice were evaluated. The PI3 kinase p85 (PI3K) inhibitor was added into the conventional mesenchymal stem cell cultures *in vitro* to observe its effects on the secretion of anti-inflammatory cytokines.

**Results:** Mesenchymal stem cells treated with poly(I:C) (anti-inflammatory phenotype) could effectively reduce serum creatinine and blood urea nitrogen, attenuate histopathological damage and apoptosis level, decrease the level of circulating pro-inflammatory cytokines and increase the level of circulating anti-inflammatory cytokines, enhance peroxidase activity and reduce malondialdehyde content at each time point. After the addition of the PI3K inhibitor, the mRNA expression and protein secretion of indoleamine 2,3-dioxygenase 1 and heme oxygenase 1 of various mesenchymal stem cells were significantly reduced, and that of mesenchymal stem cells treated with poly(I:C) (anti-inflammatory phenotype) was more obvious.

**Conclusions:** Polyriboinosinic-polyribocytidylic acid (poly[I:C]), a synthetic double-stranded RNA, whose pretreatment induces mesenchymal stem cells to differentiate into the anti-inflammatory phenotype. Anti-inflammatory mesenchymal stem cells induced by poly(I:C) can better protect renal function, alleviate tissue damage, reduce circulating inflammation levels and enhance antioxidant capacity, and achieve stronger anti-inflammatory effects through the TLR3/PI3K pathway.

## Introduction

Acute kidney injury (AKI) is a severe global challenge. Every year, more than 13 million people worldwide suffer from acute kidney injury, and nearly 10% of patients die from acute kidney injury and its complications (1, 2). Asia is the largest and most populous continental plate, with huge variations in socioeconomic development, international status, and levels of health care among various countries and regions. In addition, developing countries have a higher incidence of acute kidney injury (3). Due to the complexity and diversity of pathogenic causes and pathogenesis of acute kidney injury (3, 4), pharmacological treatment or short-term renal replacement therapy can only be adopted, but the cure rate of patients is >50% (5). Thus, acute kidney injury is not only a major problem facing the global public health field, but also a heavy medical burden to our society and families.

Ischemia-reperfusion injury is undoubtedly the most important cause of acute kidney injury (3, 6). The pathophysiological processes of acute kidney injury caused by ischemia-reperfusion injury mainly include oxidative stress injury, apoptosis of renal tubular epithelial cells, renal hemodynamic changes, glomerular endothelial microvascular dysfunction and inflammatory immune response, while excessive inflammatory reaction and immune response are considered as the most critical pathogenic link in ischemia-reperfusion injury (7, 8).

Mesenchymal stem cells (MSCs) were first identified in 1966 by Friedenstein et al. (9), and can be derived from the vast majority of human tissues, such as bone marrow, adipose and placental tissues. Because of its powerful multilineage differentiation potential, it provides a new therapeutic strategy and treatment for many clinical diseases, such as organ transplantation, autoimmune diseases and tumor diseases (10).

Mesenchymal stem cells have strong and unique immunomodulatory effects. They can be divided into two subsets, namely, pro-inflammatory phenotype and anti-inflammatory phenotype, which can also be called MSC I and MSC II (11–13). When the immune system is not fully activated, mesenchymal stem cells can promote the inflammatory response, while when the immune system is overactivated, mesenchymal stem cells can avoid excessive self-attack. This bidirectional regulatory mechanism is known as the “sensor and converter of the immune system”. Existing studies have shown that when the content of inflammatory factors in the local microenvironment is low, mesenchymal stem cells tend to differentiate into MSC I that promotes inflammation, i.e., pro-inflammatory mesenchymal stem cells. On the contrary, when it is in the microenvironment of relatively high concentrations of inflammatory factors, mesenchymal stem cells tend to differentiate into MSC II that inhibits inflammation, i.e., anti-inflammatory mesenchymal stem cells (11–13). However, the distinction between these two subgroups has not been found in their cellular phenotypes. Many studies have shown that this classification is functional and can be identified by the differences in their expression products. For example, anti-inflammatory mesenchymal stem cells usually highly express indoleamine 2,3-dioxygenase (IDO).

In addition, mesenchymal stem cells can also sense different inflammatory factor signals through Toll-like receptors (14–18). The activation of different types of Toll-like receptors (Toll-like receptor 3 or Toll-like receptor 4) can also induce different mesenchymal stem cells subsets (14–16). For example, activation of Toll-like receptor 4 (treated with lipopolysaccharide) drives the differentiation of mesenchymal stem cells toward a pro-inflammatory phenotype, whereas activation of Toll-like receptor 3 (treated with polyinosinic-polycytidylic acid) drives the differentiation of mesenchymal stem cells toward an anti-inflammatory phenotype (19, 20).

Polyriboinosinic-polyribocytidylic acid (poly[I:C]) is a synthetic double-stranded RNA (dsRNA) analog and it is a molecular pattern associated with viral infection (21). Poly(I:C) can be recognized by TLR3 and induce the activation of NF-kB and type I interferon, and the production of inflammatory cytokines or chemokines. After being activated by poly (I:C), TLR3 can effectively participate in the generation of protective immunity against some virus infections, and also participate in the activation of natural killer cells and cytotoxic T lymphocytes by myeloid dendritic cells (21). This is important for both innate and adaptive immunity. Ribes Sandra et al. found that poly(I:C) pretreatment could effectively increase the number of NK cells and regulate the innate immune response. At the same time, it can enhance the resistance of neutropenic mice to E. coli K1 meningoencephalitis (22). Du Yanqin et al. found that the use of poly(I:C) in the chronic HBV replication mouse model could increase the production of cytokines and chemokines in liver, enhance T cell response, and significantly reduce the HBsAg, HBeAg and HBV DNA levels in mice, and play a great role in anti-hepatitis B virus (23). These indicate that poly(I:C) plays a vital role in resisting bacteria and viruses, and we would like to further explore whether it can work synergistically with mesenchymal stem cells.

There have now been reports of mitigation of acute kidney injury by treatment with mesenchymal stem cells, but mesenchymal stem cells are only isolated and purified, without subdividing them into different subpopulations. In this study, untreated and different subsets of bone marrow mesenchymal stem cells were used to treat the renal ischemia-reperfusion injury model in mice, and samples were taken at different times after injury to observe the effects. Subsequently, the PI3K inhibitor, i.e., blocking TLR3/PI3K pathway, was added into the cell culture *in vitro*, and its effect on inflammatory factors secretion of different subsets of mesenchymal stem cells were observed.

## Methods

### Culture and Induce Differentiation of Mesenchymal Stem Cells *in vitro*

In this study, mouse bone marrow-derived mesenchymal stem cells (CP-M131, Procell Life Science & Technology Co., Ltd.) were divided into three groups, namely, an untreated mesenchymal stem cell group, a lipopolysaccharide-treated mesenchymal stem cell group and a polyinosinic-polycytidylic acid-treated mesenchymal stem cell group. Equal volume of common cell culture medium, 10 μg/mL lipopolysaccharide solution and 1 mg/mL polyinosinic-polycytidylic acid solution were added respectively, and after continuous culture for 24 h, cell supernatant and cell precipitate were collected and lysed. Enzyme-linked immunosorbent assay (ELISA) was used to test the content of prostaglandin *E*_2_ in cell supernatant and indoleamine 2,3-dioxygenase in cell lysates, so as to identify different subpopulations of mesenchymal stem cells. On the basis of the above results, a new group was added for each treatment method. LY294002 (PI3K pathway inhibitor, HY-10108, MedChemExpress) was added before the inducer was added, and the mRNA expressions of indoleamine 2,3-dioxygenase 1 and heme oxygenase 1 in cells were detected by real-time quantitative PCR. The protein secretion of indoleamine 2,3-dioxygenase and heme oxygenase 1 in cells were detected by western blot assay.

### Animal Model and Treatment

In this study, SPF grade male C57BL/6 mice (6–8 week old, 20–22g, purchased from Zhongshan Hospital, Shanghai, China) were used to establish a renal ischemia-reperfusion injury model. Animal procedures were conducted according to the Center for Experimental Animals of Zhongshan Hospital of Fudan University Guidelines and were approved by the Animal Care and Use Committee of Zhongshan Hospital of Fudan University, which was in accordance with the Guide for the Care and Use of Laboratory Animals published by the US National Institutes of Health. The modeling method is to use arteriole clamp to clamp the bilateral renal pedicles of the mice for 35 min and then release the pedicles, and observe the changes of kidney colors to ensure the successful modeling. We set up a sham operation group. Meanwhile, 1 mL normal saline, 1 mL untreated mesenchymal stem cells (10^7^ cells), 1 mL lipopolysaccharide-treated mesenchymal stem cells (10^7^ cells), and 1 mL polyinosinic-polycytidylic acid-treated mesenchymal stem cells (10^7^ cells) were injected into the tail vein of the mice, respectively. The mice were sacrificed and sampled at 24, 48, and 72 h after injury (*n* = 6 per group).

### Enzyme-Linked Immunosorbent Assay (ELISA)

ELISA kits were used to detect serum creatinine and urea nitrogen content to assess renal function, serum IFN-γ/IL-17/IL-10/IL-4 content to assess circulating inflammation level, and kidney glutathione peroxidase and malondialdehyde content to assess tissue antioxidant capacity.

The blood from IRI mice was obtained by heparin-coated syringe and centrifuged 3,000 rpm/min for 10 min. Supernatants were collected to quantify the serum creatinine, urea nitrogen, and IFN-γ/IL-17/IL-10/IL-4 with mouse creatinine ELISA kit (ml037726, Shanghai Enzyme-linked Biotechnology Co., Ltd.), mouse urea nitrogen ELISA kit (ml016913, Shanghai Enzyme-linked Biotechnology Co., Ltd.), mouse IFN-γ ELISA kit (ml002277, Shanghai Enzyme-linked Biotechnology Co., Ltd.), mouse IL-17 ELISA kit (ml037866, Shanghai Enzyme-linked Biotechnology Co., Ltd.), mouse IL-10 ELISA kit (ml037873, Shanghai Enzyme-linked Biotechnology Co., Ltd.) and mouse IL-4 ELISA kit (ml063156, Shanghai Enzyme-linked Biotechnology Co., Ltd.) according to the protocol. Kidney tissue was transferred to a tissue homogenizer for homogenization and centrifuged 5,000 rpm/min for 15 min. Supernatants were collected to quantify the kidney glutathione peroxidase and malondialdehyde with mouse glutathione peroxidase ELISA kit (ml058194, Shanghai Enzyme-linked Biotechnology Co., Ltd.) and mouse malondialdehyde assay kit (A003-1-2, Nanjing Jiancheng Bioengineering Institute) according to the protocol.

### Histomorphological Evaluation

Hematoxylin and eosin staining was used for semi-quantitative histopathological scoring of kidney tissues. TUNEL assay was used to detect the level of apoptosis in renal tubular epithelial cells.

The unilateral kidney (*n* = 6/group) was sliced into two equal sections. The specimens were fixed in 4% paraformaldehyde, enclosed in paraffin, stained with hematoxylin and eosin (BA-4025, Baso) according to the standard protocols, and examined by light microscopy for histology changes. The thickness of the sections used for histology stains was 4 μm. A scoring system was used to evaluate the histological renal damage. Simply, 10 fields of view of the outer medulla were taken out under 20 times of microscope for each section, and then normal = 0, slight injury = 1 (damaged renal tubules <5%), mild injury = 2 (5–25% of damaged renal tubules), moderate injury = 3 (25–75% of damaged renal tubules), severe injury = 4 (damaged renal tubules >75%). Semi-quantitative analysis was performed and the mean value was calculated as the scoring index of tubular necrosis. TUNEL immunofluorescence staining was used to evaluate and analyze the apoptosis level of renal tubular epithelial cells. According to the protocol, staining was performed using *in situ* Cell Death Detection Kit, Fluorescein (11684795910, Roche). The nuclei were blue with DAPI staining, and the apoptotic cells were green for TUNEL positive. The number of DAPI and TUNEL positive cells was counted. The percentage of TUNEL-positive cells was calculated as follows: green/blue ^*^100%.

### Real-Time Quantitative PCR

The mRNA expression of heme oxygenase 1 (HO-1), indoleamine 2,3-dioxygenase 1 (IDO-1), interleukin 1β (IL-1β) and interleukin 6 (IL-6) in cultured mesenchymal stem cells according to the experimental procedures were detected by real-time quantitative PCR.

Collecting the cells treated according to the experimental protocol. Total RNA was extracted from mesenchymal stem cell using Trizol (15596026, Invitrogen) according to the instruction. RNA was reverse-transcribed from 2 μg of total RNA by 200U of M-MuLV reverse transcriptase. Real-time PCR was carried out on a 96-well plates using AceQ Universal SYBR qPCR Master Mix (Q511-02, Vazyme) and performed with diluted cDNA using primers for heme xygenase-1 (HO-1), indoleamine 2,3-dioxygenase (IDO), interleukin-1β (IL-1β) and interleukin-6 (IL-6). A threshold cycle value (CT) was estimated using the ΔΔCT method to quantify the assessment of gene expression.

mRNA expression of the target genes was detected using the following primers: HO-1 fwd 5′-acagaagaggctaagaccgc-3′ and rev 5′-caggcctctgacgaagtgac-3′, IDO-1 fwd 5′-gttgggcctgcctcctattc-3′ and rev 5′-aagaagcccttgtcgcagtc-3′, IL-1β fwd 5′-gcaactgttcctgaactcaact-3′ and rev 5′-atcttttggggtccgtcaact-3′, IL-6 fwd 5′-tagtccttcctaccccaatttcc-3′ and rev 5′-ttggtccttagccactccttc-3′, β-actin fwd 5′-ctgagagggaaatcgtgcgt-3′ and rev 5′-ccacaggattccatacccaaga-3′. All gene expression values were normalized to the housekeeping gene β-actin.

### Western Blot

The protein secretion of PI3 kinase p85 (PI3K), phospho-PI3K (p-PI3K), heme oxygenase 1 (HO-1) and indoleamine 2,3-dioxygenase (IDO) in cultured mesenchymal stem cells according to the experimental procedures were detected by western blot assay.

Adherent cells were scraped from the culture dishes, and total protein was extracted from the cell pellet with RIPA lysis buffer (89,900, Thermo Scientific). The proteins were separated through a 12% sodium dodecyl sulfate-polyacrylamide gel electrophoresis and transferred to polyvinylidene difluoride membranes (ISEQ00010, Merck Millipore). The membrane was blocked and probed with primary antibodies overnight, including PI3 kinase p85 (PI3K, ab133595, abcam), phospho-PI3K (p-PI3K, ab182651, abcam), heme oxygenase 1 (HO-1, ab68477, abcam), indoleamine 2,3-dioxygenase (IDO, ab277522, abcam) and β-Actin (ab8226, abcam) at 1:1,000 dilutions. The membrane was then incubated with the secondary antibodies (115-035-003, Jackson). The signals were scanned for the densitometry and the final blots were quantified with ImageJ Version 2.1.0/1.53c software.

### Statistics

IBM SPSS Statistics R26.0.0.0 64-bit statistical software was used to process the experimental data, and all the measurement data in the study were expressed as mean ± standard deviation. Before further analysis of the statistical difference, normality test and homogeneity test of variance were required. After both tests were passed, the one-way analysis of variance was used to determine the statistical difference, and the pairwise *post-hoc* comparison was performed for the difference between groups using Tukey test. All the groups in the study were taken into account in the statistics, and the test efficiency was automatically adjusted according to the number of tests, so it could be guaranteed that the multiple inter-group comparisons would not increase the probability of the type I error. *P* < 0.05 indicated that the test had significant statistical difference. All statistical histograms were plotted using GraphPad Prism Version 9.0.1(128) software.

## Results

### Anti-inflammatory Mesenchymal Stem Cells Induced by Poly(I:C) Can Effectively Protect the Renal Function and Reduce the Pathological Damage in Mice With Acute Kidney Injury

**Figure 1:**
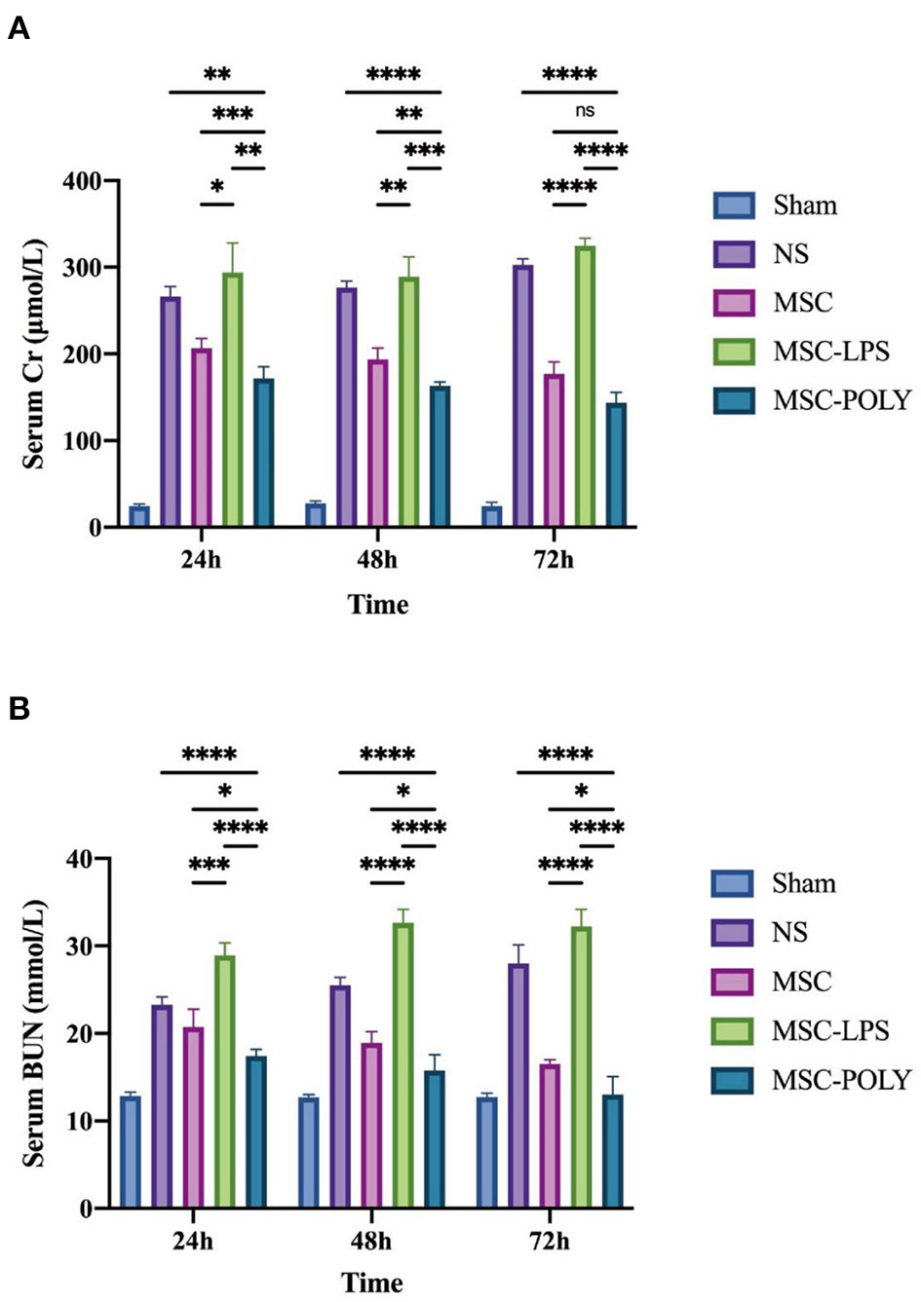
Determination of serum creatinine and blood urea nitrogen contents. **(A)** Serum creatinine contents of mice at three time points after sham operation (Sham), treatment with normal saline (NS), untreated mesenchymal stem cells (MSC), mesenchymal stem cells treated with 10 μg/mL LPS (MSC-LPS), and mesenchymal stem cells treated with 1 mg/mL poly(I:C) (MSC-POLY), respectively. **(B)** Blood urea nitrogen contents of mice at three time points of five groups. Values were expressed as mean ± standard deviation (mean ± SD), *n* = 6, *ns* = not statistically significant, ^*^P < 0.05, ^**^P < 0.01, ^***^P < 0.001, ^****^P < 0.0001.

The study found that, with the passage of time after ischemia-reperfusion injury (IRI), the serum creatinine and blood urea nitrogen contents of mice in the normal saline group and the lipopolysaccharide pretreatment group gradually increased, while those in the untreated stem cell group and the poly(I:C) pretreatment group slowly decreased. The inter-group differences at different time points showed that the serum creatinine and blood urea nitrogen contents of the mice in the untreated stem cell group were significantly lower than those in the normal saline group, the control group established in this study. The content of serum creatinine and blood urea nitrogen in the poly(I:C) pretreatment group was significantly lower than that in the untreated stem cell group. The content of serum creatinine and blood urea nitrogen in the LPS pretreatment group was comparable to or slightly higher than that in the normal saline group. We believe that mesenchymal stem cells have a certain role in protecting the renal function of mice in the natural state, and the anti-inflammatory mesenchymal stem cells pretreated with poly(I:C) can enhance the protection of the renal function of mice (Figure 1).

**Figure 2:**
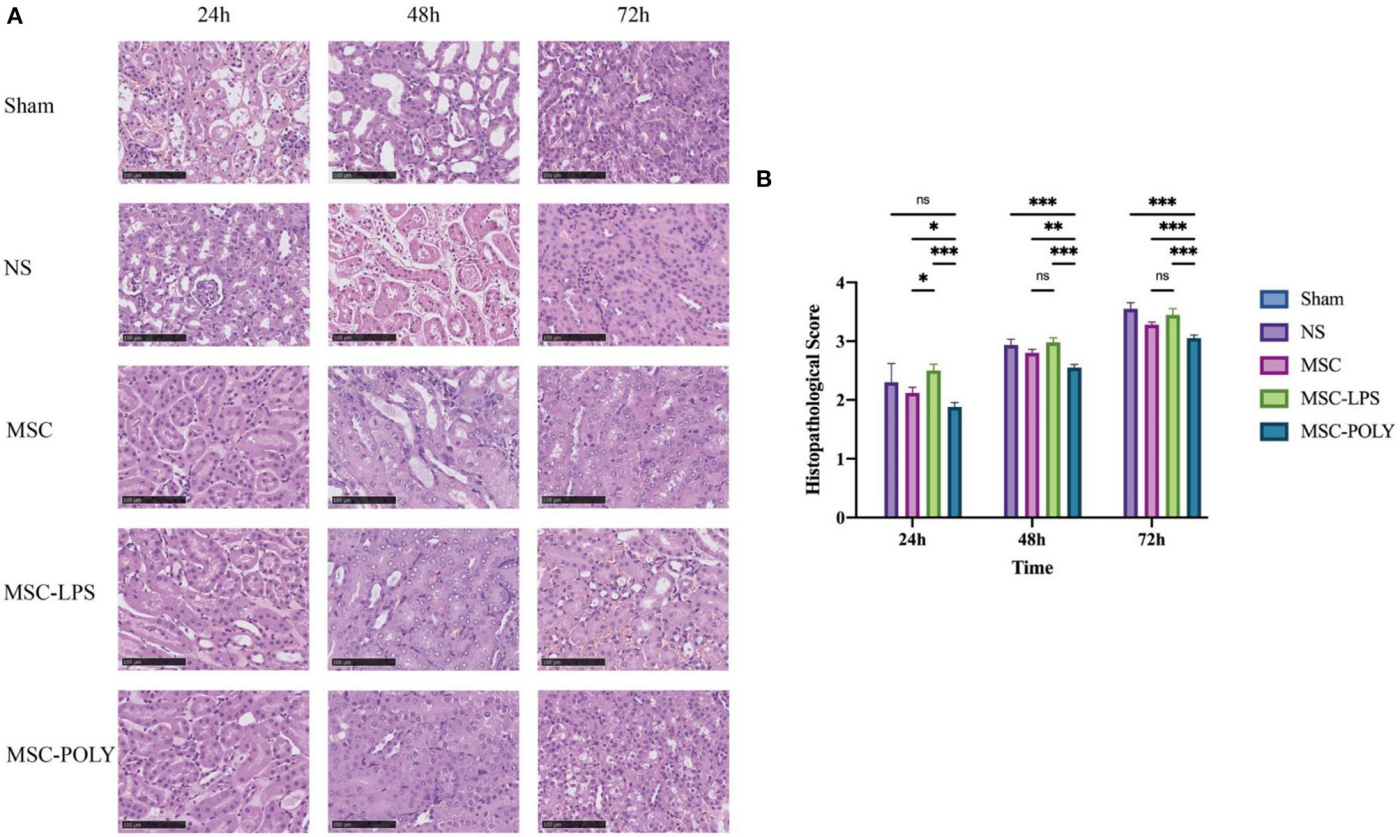
Pathological sections and scores of kidneys in mice treated with different mesenchymal stem cell subsets. **(A)** Kidney H&E staining pictures (200×) of mice at three time points after sham operation (Sham), treatment with normal saline (NS), untreated mesenchymal stem cells (MSC), mesenchymal stem cells treated with 10 μg/mL LPS (MSC-LPS), and mesenchymal stem cells treated with 1 mg/mL poly(I:C) (MSC-POLY), respectively. **(B)** Semi-quantitative histopathological scores at three time points of five groups. Values were expressed as mean ± standard deviation (mean ± SD), *n* = 6, *ns* = not statistically significant, *P < 0.05, **P < 0.01, ***P < 0.001.

It was found that the degree of degeneration and necrosis of tubular epithelial cells in all groups increased over time after ischemia-reperfusion injury, and mainly included the extent of turbid and swollen tubular epithelial cells, the proportion of renal tubular epithelial cells with vacuoles or coagulative necrosis and shedding, the degree of glomerular lesions and interstitial edema and casts seen in the renal tubular lumen, and the infiltration of inflammatory cells in the interstitium. Details of the histological semi-quantitative scoring have been listed in the “Methods” section. The renal pathological injury of mice in the untreated stem cell group was significantly milder than that in the normal saline group at each time point, while the damage in the poly(I:C) pretreatment group was comparable to or milder than that in the untreated stem cell group, while the damage in the LPS pretreatment group was comparable to or slightly severer than that in the normal saline group. At the same time, as time went by (mainly at 48 h and 72 h), the mice in poly(I:C) pretreatment group exhibited more significant alleviation of renal pathological injury, which was more obvious relative to the advantages in the other groups. The untreated mesenchymal stem cells had been able to spontaneously show a certain effect of inhibiting inflammation and delaying histopathological damage of kidney at 72 h. Overall, pathological lesions occurred at a later time point than the deterioration of renal function (Figure 2).

**Figure 3:**
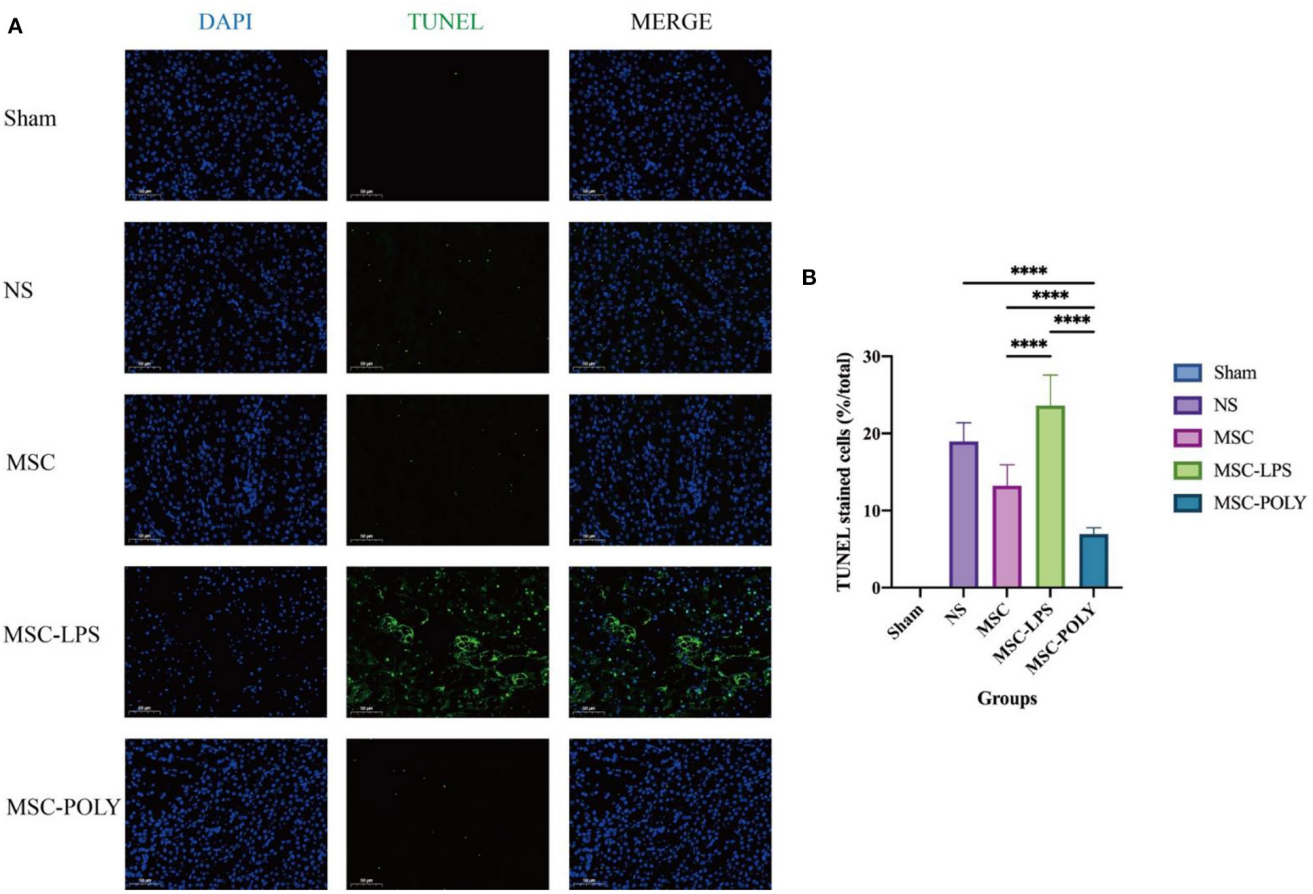
Apoptotic levels of renal tubular epithelial cells in mice treated for 72 h. **(A)** Apoptosis pictures detected by TUNEL assay (200×) at 72 h of five groups. **(B)** Proportion of TUNEL staining positive cells (i.e., apoptotic mouse renal tubular epithelial cells) at 72 h of five groups. Values were expressed as mean ± standard deviation (mean ± SD), *n* = 6, ****P < 0.0001.

At the same time, we found that the proportion of apoptotic renal tubular epithelial cells in the normal saline group was about 20%, and the apoptotic conditions were improved in both the untreated stem cell group and the anti-inflammatory mesenchymal stem cell group. The proportion of apoptotic cells in the poly(I:C) pretreatment group was the lowest, while the proportion of apoptotic cells in the LPS pretreatment group was significantly higher than that in the normal saline group (Figure 3).

### Anti-inflammatory Mesenchymal Stem Cells Induced by Poly(I:C) Can Effectively Reduce the Level of Circulating Inflammation and Enhance Their Antioxidant Capacity in Mice With Acute Kidney Injury

We selected two major types of cytokines, namely pro-inflammatory factors (IFN-γ, IL-17) and anti-inflammatory factors (IL-10, IL-4). The results of pro-inflammatory factors suggested that, with the passage of time after ischemia-reperfusion injury, the levels of normal saline group and LPS pretreatment group increased gradually, while the levels of untreated stem cell group and poly(I:C) pretreatment group decreased slowly. The contents of pro-inflammatory factors in the untreated stem cell group were significantly lower than those in the normal saline group at each time point, and those in the poly(I:C) pretreatment group were significantly lower than those in the untreated stem cell group, whereas those in the LPS pretreatment group were significantly higher than those in the normal saline group. The amount of IL-17, mainly secreted by Th17 cells, did not show significant statistical difference between the untreated stem cell group and the poly(I:C) pretreatment group, considering that mesenchymal stem cells can indirectly or directly inhibit the activation and function of Th17 cells and the underlying mechanism is relatively complex.

The results of anti-inflammatory factors suggested that, with the passage of time after ischemia-reperfusion injury, the levels of untreated stem cell group and poly(I:C) pretreatment group showed a stepwise upward trend, whereas the level of normal saline group showed a slow downward trend. The results of the LPS pretreatment group were variable (a slow rise in IL-10 and a stepwise decline in IL-4), but the overall showed a downward trend, promoting the progress of the inflammatory response.

**Figure 4:**
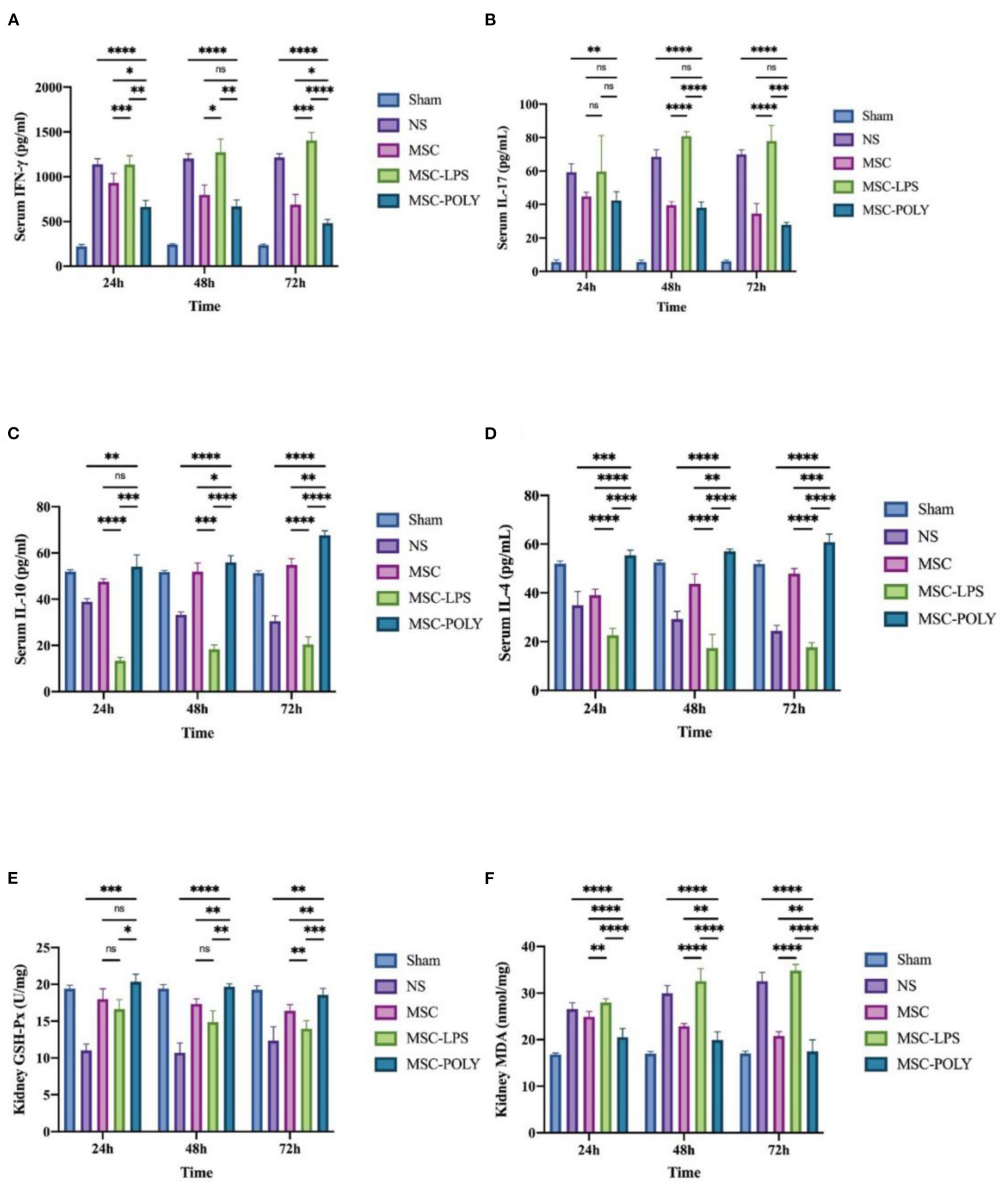
Determination of serum circulating inflammatory factors and renal glutathione peroxidase and malondialdehyde contents of mice treated with different mesenchymal stem cell subsets. **(A)** Serum IFN-γ content of mice at three time points after sham operation (Sham), treatment with normal saline (NS), untreated mesenchymal stem cells (MSC), mesenchymal stem cells treated with 10 μg/mL LPS (MSC-LPS), and mesenchymal stem cells treated with 1 mg/mL poly(I:C) (MSC-POLY), respectively. **(B)** Serum IL-17 contents of mice of mice at three time points of five groups. **(C)** Serum IL-10 content of mice at three time points of five groups. **(D)** Serum IL-4 contents of mice at three time points of five groups. **(E)** Kidney glutathione peroxidase content of mice at three time points of five groups. **(F)** Kidney malondialdehyde content of mice at three time points of five groups. Values were expressed as mean ± standard deviation (mean ± SD), *n* = 6, *ns* = not statistically significant, ^*^P < 0.05, ^**^P < 0.01, ^***^P < 0.001, ^****^P < 0.0001.

Meanwhile, we also detected the content of glutathione peroxidase as well as malondialdehyde in the kidneys of mice, and found that the glutathione peroxidase content of the untreated stem cell group and the poly(I:C) pretreatment group was significantly higher than that in the normal saline group and the LPS pretreatment group, among which poly(I:C) pretreatment group was the highest. The results of malondialdehyde content showed that with the passage of time after ischemia-reperfusion injury, the normal saline group and LPS pretreatment group showed a gradual increase, whereas the untreated stem cell group and poly(I:C) pretreatment group showed a slow decline. The malondialdehyde content in the untreated stem cell group was significantly lower than that in the normal saline group at each time point, while that in the poly(I:C) pretreatment group was significantly lower than that in the untreated stem cell group. The malondialdehyde content in the LPS pretreatment group was comparable to or higher than that in the normal saline group (Figure 4).

### Anti-inflammatory Mesenchymal Stem Cells Exert Immunosuppressive Effect Through TLR3/PI3K Pathway

Based on the above results, we found that the protective advantage of anti-inflammatory mesenchymal stem cells induced by poly(I:C) was shrunk compared with that of the remaining groups at different time points, and it was speculated that mesenchymal stem cells were still affected by the inflammatory environment *in vivo* except for the artificial intervention *in vitro*.

It is generally believed that phosphorylated PI3K/PI3K (p-PI3K/PI3K) represents the degree of PI3K activation. Western blotting showed that the activation of PI3K in LPS-pretreated MSCs was lower than that in untreated MSCs, and PI3K activation was the highest in poly(I:C)-pretreated MSCs. After the addition of LY294002, PI3K activation was reduced in all groups, most notably in the poly(I:C) pretreatment group, but it was still higher than in the other two groups (without the addition of LY294002). This indicates that, in the present study, LY294002 was added during cell culture to exert a significant PI3K inhibition and block the TLR3/PI3K signaling pathway (**Figure 6**).

**Figure 5:**
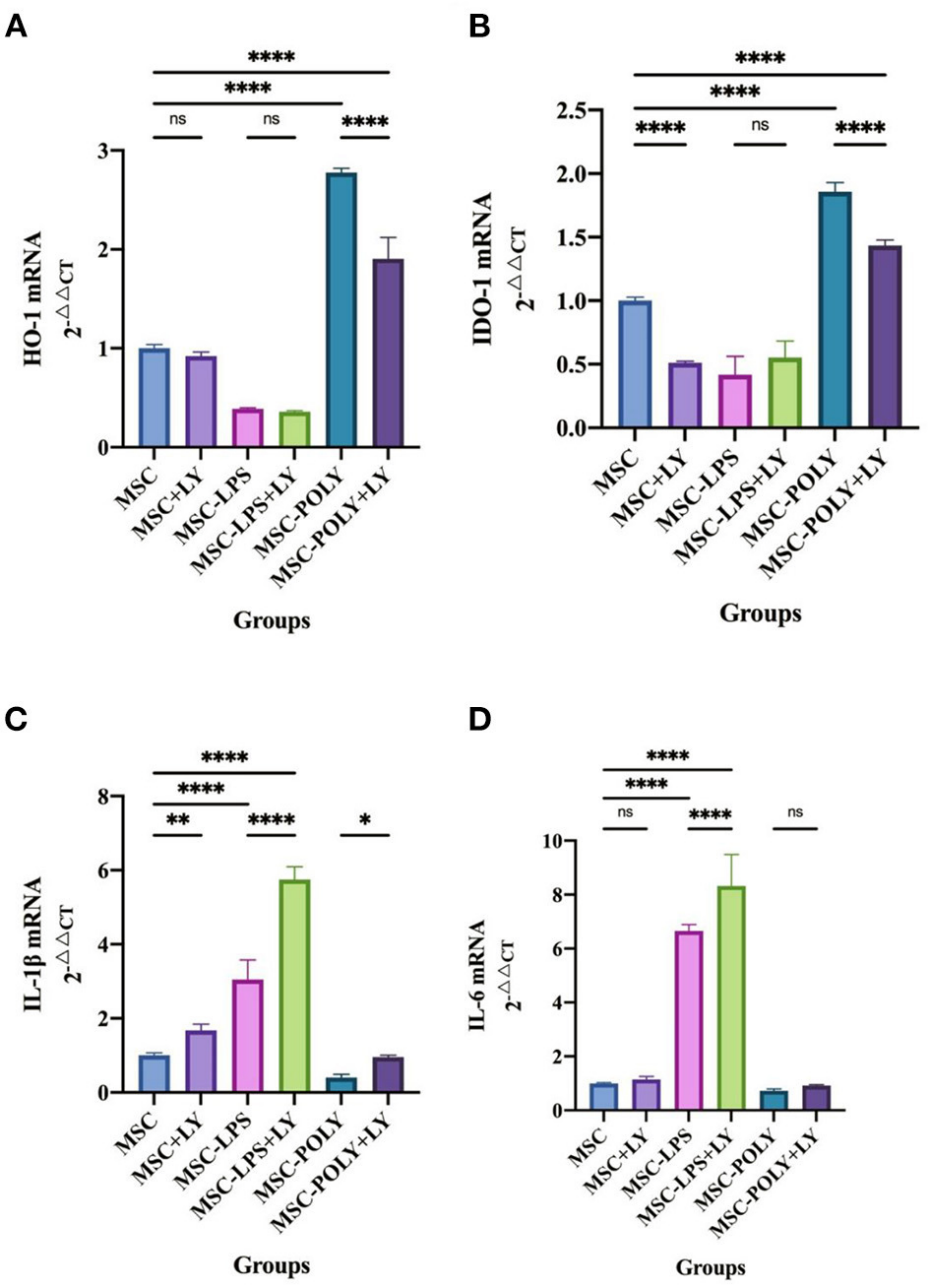
mRNA expression of cytokines in mesenchymal stem cells after addition of the PI3K inhibitor. **(A)** HO-1 mRNA expression in mesenchymal stem cells of each experimental group. **(B)** IDO-1 mRNA expression in mesenchymal stem cells of each experimental group. **(C)** IL-1β mRNA expression in mesenchymal stem cells of each experimental group. **(D)** IL-6 mRNA expression in mesenchymal stem cells of each experimental group. Values were expressed as mean ± standard deviation (mean ± SD), *n* = 6, *ns* = not statistically significant, *P < 0.05, **P < 0.01, ****P < 0.0001.

*In vitro*, we added LY294002 (a proven potent inhibitor of the TLR3/PI3K pathway) to the cell induction culture and examined the content of pro-inflammatory cytokines and anti-inflammatory cytokines secreted by mesenchymal stem cells. Real-time quantitative PCR showed that the anti-inflammatory cytokines (HO-1, IDO-1) secreted by LPS-pretreated MSCs were significantly lower than those of the untreated MSCs, while poly(I:C)-pretreated MSCs secreted the most. After the addition of LY294002, the secretion of anti-inflammatory cytokines was inhibited in all groups, most notably in the poly(I:C) pretreatment group, but it was still higher than the other two groups (without the addition of LY294002). The results of pro-inflammatory cytokines (IL-1β, IL-6) showed that the secretion amount of poly(I:C)-pretreated MSCs was slightly lower than that of untreated MSCs, and the secretion amount of LPS-pretreated MSCs was the most. After the addition of LY294002, the secretion of pro-inflammatory cytokines was increased in all groups, and the most significant one was in the LPS pretreatment group (Figure 5).

**Figure 6:**
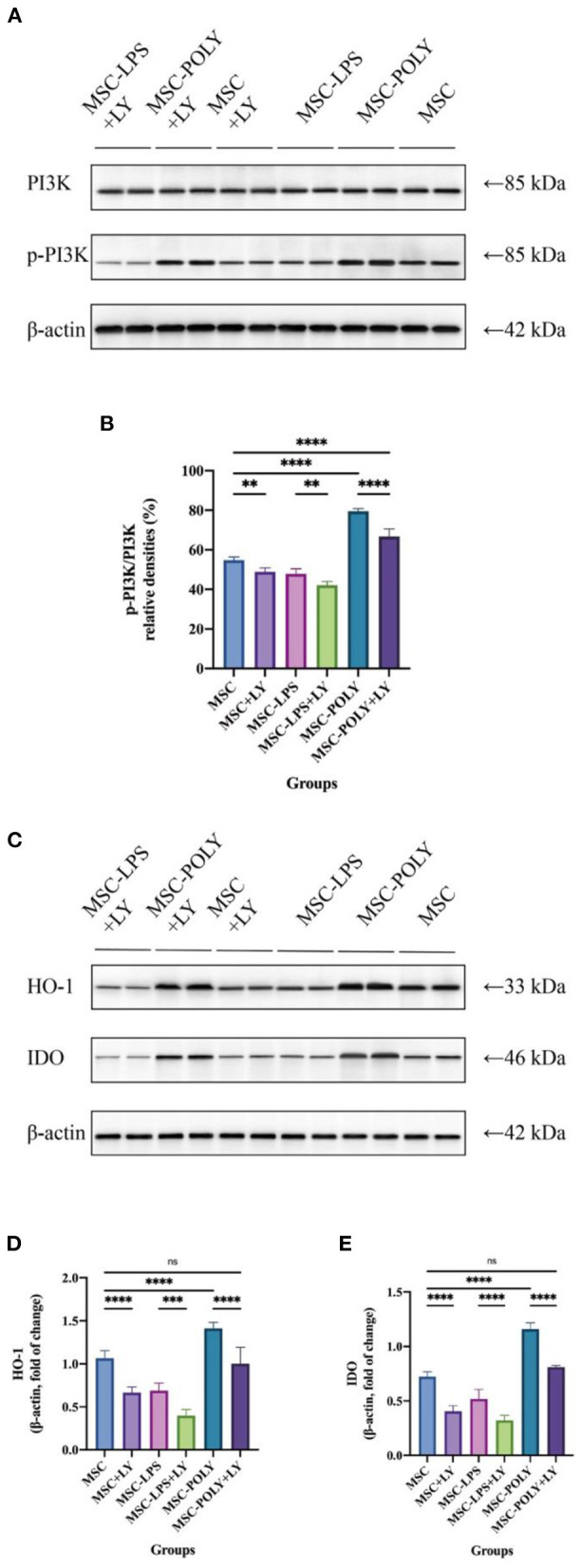
Inhibition of PI3K and protein secretion of IDO-1 and HO-1 in mesenchymal stem cells after addition of the PI3K inhibitor. **(A,B)** Representative western blot **(A)** and inhibition of PI3K **(B)** in mesenchymal stem cells of each experimental group. **(C–E)** Representative western blot **(C)** and average data for HO-1 **(D)** and IDO **(E)** in mesenchymal stem cells of each experimental group. Values were expressed as mean ± standard deviation (mean ± SD), *n* = 6, *ns* = not statistically significant, **P < 0.01, ***P < 0.001, ****P < 0.0001.

Western blotting showed that under common circumstances, HO-1 and IDO expression in lipopolysaccharide pretreated group was lower than that in untreated MSCs, while the expression in poly(I:C) pretreated group was the highest. Addition of LY294002 significantly decreased the expression of anti-inflammatory cytokines in all groups, and the secretion of anti-inflammatory cytokines by MSCs treated with poly(I:C) added inhibitor was similar to that of the untreated group (Figure 6).

## Discussion

Acute kidney injury is a common clinical complication and comorbidity, which has caused a heavy medical burden to society and families due to its complex pathogenic causes and the lack of effective treatment (1–4, 24). Previous studies have shown that renal ischemia-reperfusion injury is the most important cause of acute kidney injury (3, 6). Therefore, in this study, the mouse kidney ischemia-reperfusion model was constructed by loosening the arteriole clamp 35 min after the bilateral renal pedicles were clamped. The previous experiments confirmed that this modeling method was stable and reliable, and the survival rate of mice was high. The significant histopathological damage as well as significant increase in serum creatinine and blood urea nitrogen contents could be observed at 24, 48, and 72 h after injury.

Samples were collected at 24, 48, and 72 h throughout the study. Serum creatinine and blood urea nitrogen contents were the two gold standards most commonly used clinically to evaluate renal function. The results showed that the normal saline group and the pro-inflammatory mesenchymal stem cell group exhibited a direct ratio with time, while the untreated stem cell group and the anti-inflammatory mesenchymal stem cell group exhibited an inverse ratio with time. Mesenchymal stem cells with the effect of inhibiting inflammation can effectively prevent further renal tissue destruction by immune effector cells, protect kidney function, and play a therapeutic role, in which this protective and therapeutic effects were more pronounced when poly(I:C) pretreatment was administered. Mesenchymal stem cells with pro-inflammatory properties cannot restore renal function and have the potential to exacerbate kidney damage, which was more rapidly than the deterioration of kidney function under natural course. The results of the untreated stem cell group fluctuated, suggesting that mesenchymal stem cells had different phasic effects in the regulation of immune response. In the early stage of inflammation, they tended to be activated by pro-inflammatory signals, while in the middle and late stage of inflammation, they tended to be activated by anti-inflammatory signals. If we can effectively block the pro-inflammatory signaling pathway of mesenchymal stem cells when using them, we may achieve better therapeutic effects, alleviating kidney ischemia-reperfusion injury and protecting renal function.

Histopathological results showed that each experimental group exhibited higher semi-quantitative histopathological scores over time, mainly including the degree of more severe turbidity and swelling in renal tubular epithelial cells, higher proportion of renal tubular epithelial cells with vacuoles or coagulative necrosis and shedding, more casts visible in the renal tubular lumen, more significant glomerular lesions, more significant degree of interstitial edema, and more intensive infiltration of inflammatory cells in the interstitium. The comparison of the differences between the groups suggested that the injury degree was similar between the normal saline group and the pro-inflammatory mesenchymal stem cell group, but the injury in the pro-inflammatory mesenchymal stem cell group was slightly milder than that in the normal saline group at the later stage, while the untreated stem cell group and the anti-inflammatory mesenchymal stem cell group had a significantly better injury degree than the two groups mentioned above, and the anti-inflammatory mesenchymal stem cell group had the lowest score and the mildest damage. This indicated that even without any pretreatment, mesenchymal stem cells could play a certain role in the treatment of acute kidney injury in mice. However, if poly(I:C) pretreatment made them possess the anti-inflammatory phenotype, they could play a more significant role in resisting damage and protecting kidney tissue. On the contrary, if LPS pretreatment made them possess the pro-inflammatory phenotype, it not only would weaken the protection of untreated stem cells, but could even be observed that their damage was more serious than that of the natural course, considering that it may have activated a cascade immune response such that a large number of immune effector cells accelerate the destruction of renal tissue.

Apoptosis of renal tubular epithelial cells in mice can well reflect the degree of damage of renal tissues attacked by immune response. In this part, the 72 h samples with the most significant morphological difference were detected by TUNEL assay. We believe that when the cells double-stained with DAPI and TUNEL were apoptotic cells, and through counting the proportion of apoptotic cells, we found that when the immune response was inhibited, the proportion of apoptotic tubular epithelial cells was decreased, and the effect of anti-inflammatory mesenchymal stem cells was more significant. However, after further activation of the cascade immune response, the apoptosis of tubular epithelial cells was significantly enhanced and higher than that in the natural course of disease. Anti-inflammatory mesenchymal stem cells can effectively protect the renal tissue structure and reduce the damage of ischemia-reperfusion to the renal tubular epithelial cells, which is consistent with the histopathological results. It should be pointed out that after apoptosis, the cells would fall off from the renal tubules and enter the lumen, and then the actual apoptosis is underestimated in the analysis. Although there will be such a situation, we think that taking it as an auxiliary observation index, and taking it as a qualitative evaluation rather than a quantitative analysis, will have a certain supporting effect on the protection of renal tubular cells by mesenchymal stem cells.

Serum circulating inflammation level is an important dimension for measuring the immune response under stress. The results of pro-inflammatory factors (IFN-γ and IL-17) were basically similar to the aforementioned renal function indicators. The mesenchymal stem cells with the immunosuppressive effect could effectively reduce the content of circulating pro-inflammatory factors, reflecting its therapeutic effect on injury. Moreover, after poly(I:C) pretreatment, more significant therapeutic effect could be obtained. This means that when mesenchymal stem cells enter the damaged environment of mice without any treatment, they will differentiate into two different directions, which is mainly dependent on the complex inflammatory environment in mice. Under normal circumstances, in the early stage of inflammation, because of the low content of inflammatory factors, mesenchymal stem cells have no significant immunosuppressive effect or even enhance immunity. In the middle and late stage of inflammation, massive tissue destruction caused the injured cells to release inflammatory factors, mesenchymal stem cells will play its immunosuppressive role. Of course, the experimental results show that these two effects cannot be completely cut, only a general trend can be seen in the whole, with the two effects counteracting each other. Mesenchymal stem cells with pro-inflammatory phenotype can further activate immune effector cells, enhance the immune response, aggravate tissue damage, and enhance the circulating inflammation level of the body, so that the natural course of disease is reached or surpassed. It was also found here that changes in serum circulating pro-inflammatory cytokines peaked at 24 h or 48 h, significantly earlier than the appearance of histopathological lesions. The difference in IL-17 content of pro-inflammatory factors is noteworthy. After pairwise comparison, the serum IL-17 content at three time points between the anti-inflammatory mesenchymal stem cell group and the untreated stem cell group was not statistically significant. IL-17 is mainly secreted by the effector Th17 cells, and further mediates the mobilization, recruitment and activation of neutrophils, thus promoting the inflammatory response of the tissue. Mesenchymal stem cells can inhibit the activation and function of Th17 cells by direct cell contact or paracrine of cytokines (25). Mesenchymal stem cells enhance CD54 expression through the CCR6-CCL20 pathway and recruit Th17 cells to mesenchymal stem cells (26), which can inhibit the differentiation of Th17 cells by up-regulating IL-10, PD-1, CCL2 or SOCS3 (26–29), and blocking STAT3 pathway (28, 29). At the same time, when the STAT3 pathway is blocked, it can also down-regulate the expression of RORt and IL-17, thereby further inhibiting the differentiation of Th17 cells (30). But at the same time, some studies have put forward different opinions, that is, in some mixed lymphocyte experiments, mesenchymal stem cells have shown the function of promoting IL-17 secretion and stimulating the activation of Th17 cells (31). Of course, this experiment has certain limitations (31). In most other similar experiments, mesenchymal stem cells can significantly promote the proliferation and differentiation of Treg cells. However, it has not been found in this experiment, so the experimental results need to be further explored (31). In our research, it is believed that mesenchymal stem cells may have both effects. Although they reduce the content of IL-17, a pro-inflammatory factor, by inhibiting inflammation, they also partially stimulate the activation of Th17 cells, but the specific mechanism and details of action still need to be further clarified.

The results of serum anti-inflammatory factors (IL-10 and IL-4) were basically consistent with the ones mentioned above. It is noteworthy that the results of the pro-inflammatory mesenchymal stem cell group were different. The content of serum IL-10 in mice increased slowly over time, while serum IL-4 content was basically flat, suggesting that mesenchymal stem cells with pro-inflammatory phenotype treated *in vitro* might still be activated by some related anti-inflammatory signaling pathways and secrete anti-inflammatory cytokines in the complex and volatile inflammatory environment *in vivo*. Mesenchymal stem cells have the ability to switch between the two phenotypes, which is mainly dependent on the local concentration of inflammatory factors and the phase of inflammatory response. Of course, this effect is weak, only partially offsetting its pro-inflammatory effect.

One of the key pathogenic causes of acute kidney injury is cascade-amplified inflammatory response, which includes various inflammatory processes, complement activation and innate immunity mobilization (32). Oxidative stress injury is also one of the important causes, suggesting that the production of various free radicals is increased, and these generated free radicals cannot be degraded and eliminated by substances containing enzyme or non-enzyme components. Renal ischemia-reperfusion injury mainly includes membrane lipid peroxidation, protein and DNA oxidative damage, and the resulting apoptosis and necrosis (32). In this study, the glutathione peroxidase activity and malondialdehyde content were used to assess the antioxidant capacity of the mice. The results suggested that mesenchymal stem cells with anti-inflammatory phenotype could not only inhibit the immune response but also effectively increase the content and activity of peroxidase in the body and reduce the production of malondialdehyde in the tissue, thus better protecting the tissue and enhancing the antioxidant capacity of the body in response to ischemia and hypoxia injury. Mesenchymal stem cells with pro-inflammatory phenotype can aggravate oxidative stress damage, weaken the activity of peroxidase, and allow the accumulation of lipid peroxidation products in the tissue, impeding the recovery of function.

*In vitro* western blotting experiments showed that PI3K activation in the poly(I:C) pretreatment group was higher than that in the untreated group, while it was the lowest in the LPS pretreatment group. After the addition of LY294002, the activation of PI3K was significantly inhibited in all groups. Poly(I:C) pretreatment group had the most significant inhibition, but the activation of PI3K was still higher than that of the other two groups without adding LY294002. These results indicated that LY294002 mainly inhibited the TLR3/PI3K signaling pathway in this study, and this pathway also played an important role in the anti-inflammatory effect of mesenchymal stem cells.

*In vitro* cell experiments showed that both mRNA expression and protein secretion of anti-inflammatory cytokines were lower in the LPS pretreated group than in the untreated group under normal conditions, while the poly(I:C) pretreated group was the highest. Addition of a TLR3/PI3K inhibitor resulted in a significant decrease in the mRNA expression and protein secretion of pro-inflammatory cytokines in each group, and the reduction was most pronounced in MSCs treated with poly(I:C), which was already similar to that of the untreated group, indicating that it may have lost a potent pro-inflammatory function.

The mRNA expression levels of pro-inflammatory cytokines showed that the expression level of poly(I:C) pretreatment group was slightly lower than that of the untreated group, and the expression level of LPS pretreatment group was the highest. After the addition of TLR3/PI3K signaling pathway inhibitor, the expression levels of each group were increased, and the increase in the LPS pretreatment group was the most significant.

There are still some shortcomings in this study. In the animal experiment part, due to the large and complex molecules downstream of the related signal pathways, a relatively macroscopic phenomenon was observed, and further studies are needed to block the more specific signaling molecules and proteins downstream to observe their effects in detail. Meanwhile, attention should be paid to animal toxicity. In addition, in the present study, we only observed the effects and therapeutic effects of different mesenchymal stem cell subsets on acute kidney injury, but did not observe the effects on the overall survival time or other diseases of mice. Further long-term research in mouse models of different ages may be needed, such as whether anti-inflammatory mesenchymal stem cells promote tumorigenesis in older mice.

## Conclusions

Compared with the common mesenchymal stem cells, the anti-inflammatory mesenchymal stem cells induced by poly(I:C) have better effects of protecting renal function, alleviating tissue damage, reducing circulating inflammation level and enhancing oxidation resistance. The anti-inflammatory mesenchymal stem cells achieve the above strong anti-inflammatory effects through the TLR3/PI3K pathway. We still need further research to confirm which specific signaling molecules in the downstream of TLR3/PI3K play a vital role, and whether cell treatment with artificially modified mesenchymal stem cells brings about side effects.

## Data Availability Statement

The original contributions presented in the study are included in the article/supplementary material, further inquiries can be directed to the corresponding authors.

## Ethics Statement

The animal study was reviewed and approved by Animal Ethical Committee of Zhongshan Hospital, Fudan University.

## Author Contributions

CY and RR: conceived the project, designed the project, extracted and analyzed data, and approved the final manuscript. TC: drafted the manuscript. TC, YJ, SX, YC, and JW: conducted the experiments. All authors contributed to the article and approved the submitted version.

## Funding

This work was supported by the National Key R&D Program of China (2018YFA0107501 to RR), National Natural Science Foundation of China (81770747 and 81970646 to RR).

## Conflict of Interest

The authors declare that the research was conducted in the absence of any commercial or financial relationships that could be construed as a potential conflict of interest.

## Publisher's Note

All claims expressed in this article are solely those of the authors and do not necessarily represent those of their affiliated organizations, or those of the publisher, the editors and the reviewers. Any product that may be evaluated in this article, or claim that may be made by its manufacturer, is not guaranteed or endorsed by the publisher.

## Abbreviations

CCL: C-C motif ligand
CCR6: C-C motif chemokine receptor 6
CD54: cluster of differentiation 54
DAPI, 4′: 6-diamidino-2-phenylindole
ELISA: enzyme-linked immunosorbent assay
IDO: indoleamine 2,3-dioxygenase
IFN-γ: interferon gamma
IL: interleukin
mRNA: messenger RNA
MSC: mesenchymal stem cell
PCR: polymerase chain reaction
PD-1: programmed cell death protein 1
PGE2: prostaglandin E2
PI3K: phosphatidylinositol-3 kinase
Poly(I:C): polyinosinic-polycytidylic acid
RORt: retinoic acid-related orphan receptor
SOCS3: suppressor of cytokine signaling 3
SPF: specefic pathogen free
STAT3: signal transducer and activator of transcription 3
Th17 cell: T helper cell 17
TLR3: Toll-like receptor 3
TUNEL: terminal dexynucleotidyl transferase (TdT) -mediated dUTP nick end labeling.
